# RuralCovidLife: A new resource for the impact of the pandemic on rural Scotland.

**DOI:** 10.12688/wellcomeopenres.17325.2

**Published:** 2022-05-23

**Authors:** Anna J Stevenson, Charlotte F Huggins, Alison Forbes, Jim Hume, Grant Fulton, Claire Thirlwall, Janet Miles, Chloe Fawns-Ritchie, Archie Campbell, Clifford Nangle, Rebecca Dawson, Rachel Edwards, Robin Flaig, Louise Hartley, Christie Levein, Daniel L McCartney, Ian J Deary, Caroline Hayward, Riccardo E Marioni, Andrew M McIntosh, Cathie Sudlow, David J Porteous

**Affiliations:** 1 Centre for Genomic and Experimental Medicine, Institute of Genetics and Cancer, University of Edinburgh, Edinburgh, EH4 2XU, UK; 2 Support in Mind Scotland, Edinburgh, EH16 5GA, UK; 3 Fulton Fisheries Consultancies Limited, Isle of Harris, HS3 3DX, UK; 4 Directorate of Public Health, Dumfries and Galloway Council, Dumfries, DG1 2DD, UK; 5 The GALE Centre, Gairloch, IV21 2BH, UK; 6 Department of Psychology, University of Edinburgh, Edinburgh, EH8 9JZ, UK; 7 Centre for Medical Informatics, Usher Institute, University of Edinburgh, Edinburgh, EH16 4UX, UK; 8 MRC Human Genetics Unit, Institute of Genetics and Cancer, University of Edinburgh, Edinburgh, EH4 2XU, UK; 9 Division of Psychiatry, University of Edinburgh, Edinburgh, EH10 5HF, UK

**Keywords:** cohort, longitudinal study, COVID-19, rural communities

## Abstract

RuralCovidLife is part of Generation Scotland’s CovidLife project, investigating the impact of the COVID-19 pandemic and mitigation measures on people in Scotland. The RuralCovidLife project focuses on Scotland’s rural communities, and how they have been impacted by the pandemic.

During survey development, Generation Scotland consulted with people living or working in rural communities, and collaborated with a patient and public involvement and engagement (PPIE) group composed of rural community leaders. Through this consultation work, the RuralCovidLife survey was developed to assess the issues most pertinent to people in rural communities, such as mental health, employment, transport, connectivity, and local communities.

Between 14th October and 30th November 2020, 3,365 participants from rural areas in Scotland took part in the survey. Participant ages ranged from 16 to 96 (mean = 58.4, standard deviation [SD] = 13.3), and the majority of the participants were female (70.5%). Over half (51.3%) had taken part in the original CovidLife survey.

RuralCovidLife includes a subsample (n = 523) of participants from the Generation Scotland cohort. Pre-pandemic data on health and lifestyle, as well as biological samples, are available for these participants. These participants’ data can also be linked to past and future healthcare records, allowing analysis of retrospective and prospective health outcomes.

Like Generation Scotland, RuralCovidLife is designed as a resource for researchers. RuralCovidLife data, as well as the linked Generation Scotland data, is available for use by external researchers following approval from the Generation Scotland Access Committee. RuralCovidLife can be used to investigate mental health, well-being, and behaviour in participants living in rural areas during the COVID-19 pandemic, as well as comparisons with non-rural samples. Moreover, the sub-sample with full Generation Scotland data and linkage can be used to investigate the long-term health consequences of the COVID-19 pandemic in rural communities.

## Introduction

The coronavirus (COVID-19) pandemic, and the resultant infection control measures, led to drastic changes in everyday life across the world. In the United Kingdom (UK), the first national lockdown was implemented on 23 ^rd^ March 2020, resulting in major restrictions to the population’s way of living. This included workplace and school closures, strict instructions to stay at home except for very limited purposes, and the introduction of the Job Retention Scheme, placing many people in the UK on furlough for indeterminate periods of time. After the initial gradual easing of restrictions in 2020, a second lockdown was implemented in Scotland from 5 ^th^ January to 19 ^th^ July 2021.

From early in the pandemic, many have expressed concerns that such measures may have long-term consequences on mental health and well-being ^1^, and that research is needed to document and measure these effects ^2^. Subsequent longitudinal studies have found small increases in mental health difficulties during the COVID-19 pandemic compared to pre-pandemic ^3– 8^. However, effects are highly heterogeneous between studies, indicating that the impact of the pandemic may vary across populations ^5^. For instance, young people and women tended to show greater negative effects during the pandemic compared to other groups ^6– 8^. However, these studies did not closely examine differences between rural and urban settings.

In Scotland, 17% of the population live in rural locations ^9^, classed as settlements with populations of fewer than 3,000 people. Moreover, rural communities typically face different challenges compared to the rest of the population, particularly in regards to transport, access to services, and employment. Despite this, rural communities have rarely been the focus of COVID-19 research.

The small body of existing evidence suggests that while rural communities may be similarly negatively affected by COVID-19 compared to urban communities, they also face unique challenges. Work on rural communities in the United States of America (USA) suggests the pandemic has had a strong negative effect on the well-being and economic status of people in these communities ^10^. Moreover, the existing digital and health disparities between urban and rural communities may place rural residents at greater health risk from COVID-19 and the negative effects of the pandemic ^11^. Research in rural China suggests that while rural residents were less likely to report psychological distress during the pandemic, they reported qualitatively different concerns, such as transport and digital access ^12^.

These findings suggest that rural communities may face particular challenges during the COVID-19 pandemic. As such, a dataset capturing rural experiences during the pandemic would be valuable for future research, and for informing policy on rural recovery post-COVID-19. Generation Scotland developed the RuralCovidLife project to address this gap in available cohort data.

Generation Scotland is a large, longitudinal research study looking at the health and well-being of volunteers and their families across Scotland ^13^. This cohort has detailed socio-demographic, psychological, and genetic data from over 24,000 volunteers across Scotland, collected between 2006 and 2011, as well as health record linkage ^13^. From the beginning of the first national UK lockdown, Generation Scotland temporarily pivoted to conduct COVID-19 research.

In April 2020, Generation Scotland launched the CovidLife project, surveying how adults across Scotland were responding to the pandemic and subsequent COVID-19 mitigation measures ^14^. Following the success of this project, two sister projects were launched: TeenCovidLife ^15^, surveying Scottish adolescents, and RuralCovidLife, surveying people age 16 and above in rural Scotland. Rural Scotland is defined using the Scottish Government Urban-Rural Classification ^16^, which classifies settlements with a population of less than 3,000 as rural.

RuralCovidLife was designed to assess how the COVID-19 pandemic and subsequent mitigation measures affected those living in Scottish rural communities. This was done through participatory work with people living and working in rural communities. The questions were designed by, and for, people from rural communities, to give a voice to those living in rural locations and influence policy defining how they will be supported in the future. The RuralCovidLife survey ran from 14th October to 30th November 2020 and was open to anyone aged 16 or over living in rural Scotland. This paper describes the development of the survey, characterises the cohort, and summarises the data available to researchers. This is a data note intended to act as an information resource for researchers, and does not make any interpretations of the data or its implications.

## Methods

### Questionnaire development

RuralCovidLife began with the goal of adapting the CovidLife surveys for use in rural communities. To do so, Generation Scotland consulted with members of rural communities, particularly industry and community leaders, as well as rural life experts from a range of specialities and areas. Participants in the consultation and patient and public involvement and engagement (PPIE) work included representatives from organisations such as the National Rural Mental Health Forum, Seafood Scotland, National Farmers Union Scotland, and the Scottish Rural Network.

This work informed the design of the RuralCovidLife survey. Informal consultations first identified the key challenges faced by rural communities in the COVID-19 pandemic. These key themes were discussed in the PPIE group to further refine the survey and ensure it was relevant to rural communities. The PPIE group also played an important role in promoting RuralCovidLife within their communities.

PPIE members shaped the topics addressed in the survey from the beginning of the project. They also helped refine the survey to be suitable for rural communities, providing feedback on the final questionnaire. Some PPIE members also helped to co-author the current paper, providing feedback and comments on the manuscript.

### Consultation

Fourteen contacts took part in the initial consultations, all of whom were people living in rural Scottish communities and had expertise in relevant fields, such as tourism, business, or mental health. Initial informal consultations took place over the phone, during which informants were asked questions about the following topics:

- Key challenges faced by rural communities in lockdown
- Key challenges for rural communities leaving lockdown
- Main issues facing rural communities before lockdown
- The potential benefits of lockdown for rural communities

From these initial consultations, the following themes emerged as the main issues facing rural communities in the pandemic:

- Job loss and impact on the local economy
- Anxiety and frustration about tourism (e.g., re-opening of the Highlands while COVID-19 infections were still relatively high)
- Transport and accessibility
- Digital connectivity
- Under-representation of rural communities in both general and COVID-19 research and policy
- Different ways inequality manifests in rural communities compared to urban communities
- Having multiple jobs and mixed economy
- Mental health

The economic impact of lockdown emerged as one of the biggest issues for those interviewed, with many reporting that communities have been affected by changes to tourism and hospitality. Those interviewed believed that such changes could exacerbate pre-existing challenges to job security. Many of those interviewed also reported heightened anxiety in their communities with the easing of lockdown, particularly for young or elderly populations, with concerns that the re-opening of tourism would lead to higher rates of infection in rural areas. The consultation members also viewed existing issues around both private and public transport as another barrier to the recovery of rural communities post-lockdown, as well as a contributor to financial inequality.

The consultation also highlighted the resilience of rural communities. Many interviewed felt local rural communities were faster in providing necessary support than the government or local councils, indicating that rural communities may not rely on government structures to the same extent as an urban community. Moreover, consultants viewed this sense of independence and unity as a key strength of rural communities and claimed that such strength will be essential for recovering from the impact of the pandemic and associated mitigation measures.

This consultation directly informed how to adapt the CovidLife surveys for rural participants, as well as what topics needed to be introduced to reflect the priorities of rural communities in Scotland.

### Public and patient involvement and engagement groups

A key part of the development of the RuralCovidLife survey was the creation of a PPIE group and running workshops with this group. The group brought together the viewpoints and experiences of people living or working in rural communities in Scotland. This PPIE group was set up remotely and all sessions were run online due to the COVID-19 restrictions. Seven people from across Scotland took part in this PPIE group, all of whom lived in rural communities, worked in rural areas, or both.

To build rapport before the workshops, each member of the PPIE group had at least two individual calls with a member of the Generation Scotland team. These calls built trust with group members and ensured all members fully understood what their participation would involve. The group took part in several online workshops, either one-to-one with a member of the Generation Scotland team, or paired with another PPIE group member. Workshops were conducted via online video conferencing or over the phone. The format for each session was chosen based on the group member’s availability, preference, and internet access.

In these workshops, participants read the drafted questions for the RuralCovidLife survey, discussed the questionnaire and provided feedback to ensure questions authentically represented the priorities of rural communities and were inclusive to members of those communities.

Regular contact was maintained with the PPIE group, sharing information about the launch of the survey, press coverage, engagement levels, and other relevant information. They also received the initial report ^17^ before it was shared with the press and published online. All members were also offered to be authors on the current paper or to be included as a named acknowledgement. The PPIE members who elected to be part of the author team and read, commented on, and approved the manuscript, were included in the author list.

### Building the questionnaire

The RuralCovidLife questionnaire was developed by the Generation Scotland team using Qualtrics survey software ^18^ and could be completed on desktop computers, tablets, and smartphones. Due to COVID-19 restrictions, data collection for RuralCovidLife was limited to online assessments only.

Due to the potentially sensitive nature of some questions in the survey, answering each question was not compulsory. Additionally, participants could choose ‘Prefer not to answer’ for many questions. If a participant left a question unanswered, they were informed of the missing response and asked to confirm if they wanted to continue without answering. Participants could save their responses and return to the survey later to complete them. The survey took approximately 30 minutes to complete.

### Sample and recruitment

Anyone age 16 or over and living in rural Scotland was eligible to take part in the RuralCovidLife survey. While ‘rural Scotland’ was not defined in any further detail in the consent material, postcode was collected so that participant’s residence could be compared to the Scottish Rural-Urban Classification ^9^. Recruitment was conducted through convenience sampling, so the sample may not be representative.

Internet access was required to participate. Data collection commenced on Wednesday 14 ^th^ October 2020 and closed to new participants on Monday 30 ^th^ November. Any participant who had started, but not finished the survey, had 14 days to complete their responses.

Various recruitment strategies were used to enroll participants into RuralCovidLife. Email invitations were sent to 5,080 eligible participants from CovidLife, TeenCovidLife, and Generation Scotland cohort members with rural postcodes. Of these, 470 were undeliverable. Those invited who had not yet completed the survey or taken part were sent reminder emails on the 16 ^th^ November 2020. Of the 4,610 invited, 46.9% (n = 2,163) responded and were included in the final dataset.

The National Rural Mental Health Forum helped launch the RuralCovidLife survey via its 200 membership organisations by holding an online seminar on October 14 ^th^ 2020. Attendees were from the private, public and third sector, and national media promoted the launch.

The PPIE group also played a crucial role in promoting the survey within their communities, with a number taking part in TV, radio, and print media interviews at launch. Information about taking part in the study was also included in the newsletters and social media of industry networks in rural Scotland, including Scottish Rural Action, the Crofting Federation, the National Farmers’ Union of Scotland, and the Rural Youth Project.

Geo-targeted social media advertising targeted participants from rural areas in Scotland, areas under-represented in wider research and in the initial survey response, such as the North West and the Scottish borders. This includes both organic and paid-for social media.

Participants were also recruited through the Scottish Health Research Register (SHARE) ^19^. SHARE is a register of people age 11 and over in Scotland who expressed an interest in taking part in health research. After selecting those with rural postcodes, 7,190 members of their database were invited to take part.

### Questionnaire content

The RuralCovidLife questionnaire content was developed to capture how the pandemic had impacted those living in rural communities. A summary of the topics assessed in the survey is shown in Table 1. The full RuralCovidLife questionnaire is available in the *Extended data* ^20^. The Qualtrics survey file can also be requested from the authors.

**Table 1.** Summary of data collected in the RuralCovidLife questionnaire.

Sociodemographic	Age; sex; gender identity; ethnic origin; postcode; household composition; relationship status; household income; receipt of benefits; highest educational qualification; accommodation type and tenure; dependents in household; caring responsibilities
Health	Self-reported health conditions; whether contacted about shielding; whether had COVID-19 (suspected or tested); COVID-19 symptoms; healthcare access
Psychological	Depressive symptoms; anxiety symptoms; wellbeing; life satisfaction
Social support and relationships	Loneliness; isolation; whether someone could provide support if had COVID-19
Employment and finances	Employment status (prior to COVID-19 pandemic and now); employment industry; opinions on industries impacted by COVID-19 pandemic; whether furloughed; whether working from home; key worker status; concern about impact of COVID-19 on business/livelihood; application for support for business/livelihood; job security; financial situation (prior to COVID-19 pandemic and now); concern about finances
COVID-19 knowledge, attitudes and behaviour	Confidence in Scottish government to prevent further outbreaks of COVID-19; attitudes towards government guidelines for COVID-19; whether following government guidelines for COVID-19; whether or not installed the Protect Scotland (NHS Scotland Test & Protect) app
Connectivity	Internet connection type and quality; whether applied for Scottish Broadband Voucher Scheme; importance of reliable high speed broadband for work, keeping in touch with friends and family, accessing health and support services, and children’s school work
Transport and accessibility	Whether consider where they live to be rural; whether live on island; public transport use (prior to COVID-19 pandemic and now); difficulty planning routes (prior to COVID-19 pandemic and now); vehicle ownership; whether need to drive as part of job
Community	Rating of local area as place to live; feeling of belonging; community engagement (prior to COVID-19 pandemic and now); concern about community knowing about COVID-19 infection; how aspects of community life have been affected by COVID-19 measures; concern about visitors; attitudes on when tourists should be allowed to return

Psychological distress was measured with commonly used, validated scales. The nine-item Patient Health Questionnaire (PHQ-9) was used to assess depression ^21^, and anxiety symptoms were assessed with the seven-item Generalised Anxiety Disorder (GAD-7) ^22^. Loneliness, social isolation, and life satisfaction were also assessed. Other questions on topics such as transport and connectivity were drawn from the longitudinal Scottish Household Questionnaire 2018 ^23^, as well as cross-sectional studies such as the National Rural Mental Health Survey Scotland ^24^, and the Scottish Rural Action Covid Survey ^25^.

### Procedure

A personalised link to the RuralCovidLife survey was included in the email invitations, and a general link was shared on social media for the general public. The survey began with a participant information sheet outlining the aims of the survey and what involvement would entail. Following this, participants completed a consent form confirming their age, rural location, and consent to re-contact in the future. The consent and volunteer information sheet is available in the *Extended Data* ^20^. After beginning the survey, participants had 14 days to complete it. They could stop and return to the survey at any point during this time.

### Ethical considerations

RuralCovidLife was reviewed and given a favourable opinion by the East of Scotland Research Ethics Committee (Reference: 20/ES/0021 AM09).

## Results

Between 14 ^th^ October and 30 ^th^ November 2020, data was collected for RuralCovidLife in Qualtrics. Two surveys were created to gather data. The first was available to the general public and could be accessed by anyone. The second was distributed directly to invited participants from Generation Scotland or CovidLife, using personal links that allowed linkage to their GS or CovidLife data. Both the general public and linked versions of the questionnaires can be seen in the *Extended data* ^20^.

In total, 4,635 responses were recorded. Responses were recorded even if participants only opened the survey, or did not answer any of the questions.

Data cleaning was conducted separately by two researchers. Final exclusions were compared and any inconsistencies were resolved through discussion and further analysis until exclusions were identical. Criteria for inclusion are as follows:

1. Participants complete the survey after the official launch (Excluded n = 5).
2. Participants must have submitted at least one page of the survey after completing the consent procedure (Excluded n = 1,224).
3. Participants took part in the survey only once. Participants were classed as duplicates if both their names and email addresses were exactly the same. If email addresses were identical but names differed even slightly, they were classed as separate participants using a shared email address. For records from duplicate participants, the most completed record was preserved. If records had the same amount of progress through the questionnaire, the first record was taken (Excluded n = 34).
4. Participants must have answered at least one question across the survey (i.e., data must not have consisted of NA responses only; (Excluded n = 7).

Following data cleaning, 1,270 participants were excluded, leaving 3,365 participants in the final dataset.

**Figure 1.**
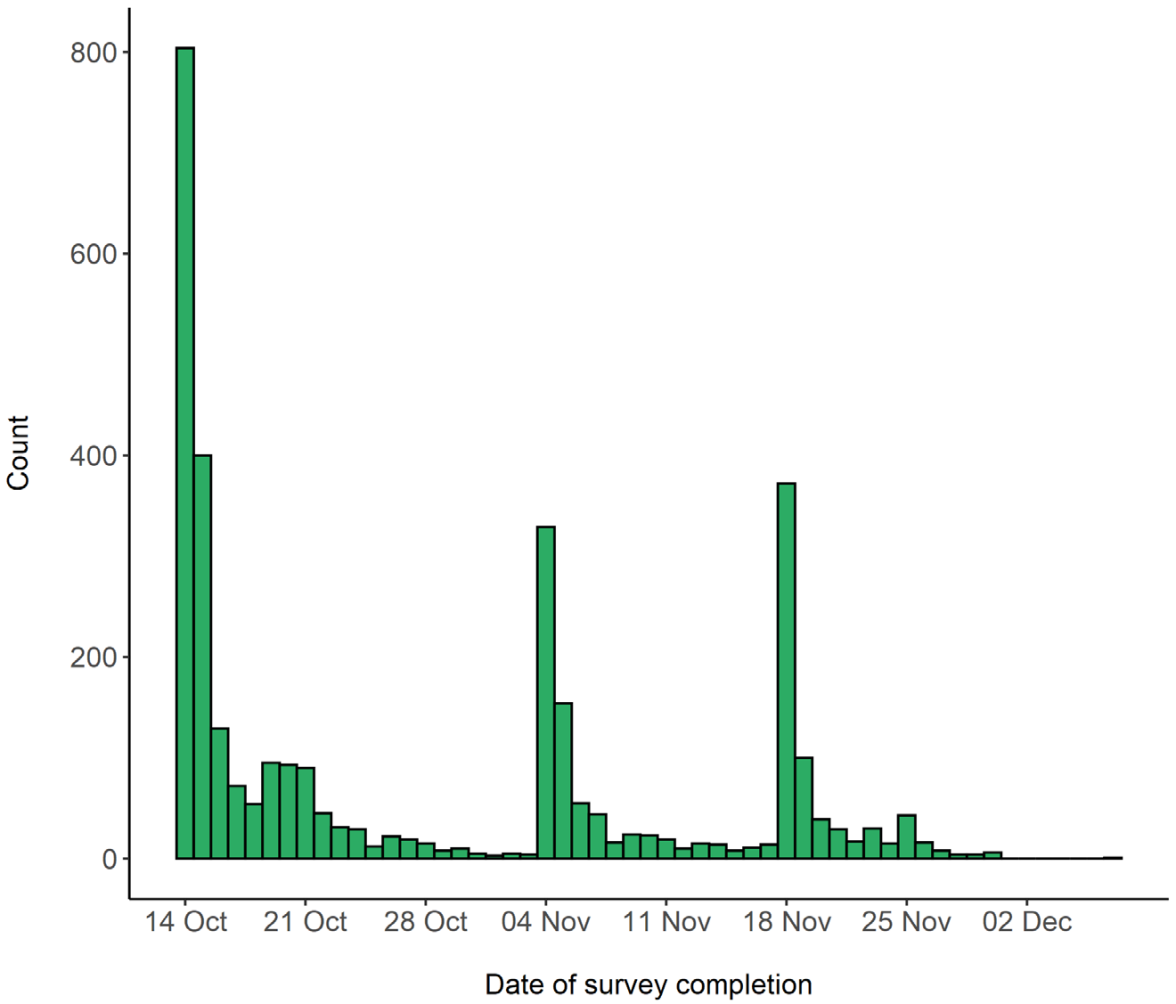
Survey completion dates for RuralCovidLife.

Figure 1 shows the completion dates of the survey. Over half of these participants had additionally taken part in the original CovidLife survey (n = 1,744, 51.8%) and 523 (15.5%) participants were members of Generation Scotland. A 0.7% (n = 23) fraction also took part in TeenCovidLife.

Scottish rural-urban classification was derived from participant postcodes, based on the Scottish Government Rural-Urban Classification ^9^. Some participants (4.4%; n = 149) were classed as living in a non-rural area, despite the survey requiring self-selection of people from rural areas. These participants were retained in the dataset as all participants confirmed their rural location in the consent, and thus their given postcode may not reflect their involvement in rural life (e.g., those who may have been temporarily relocated during data collection). However, these participants can be easily excluded from future analyses if necessary.

**Figure 2.**
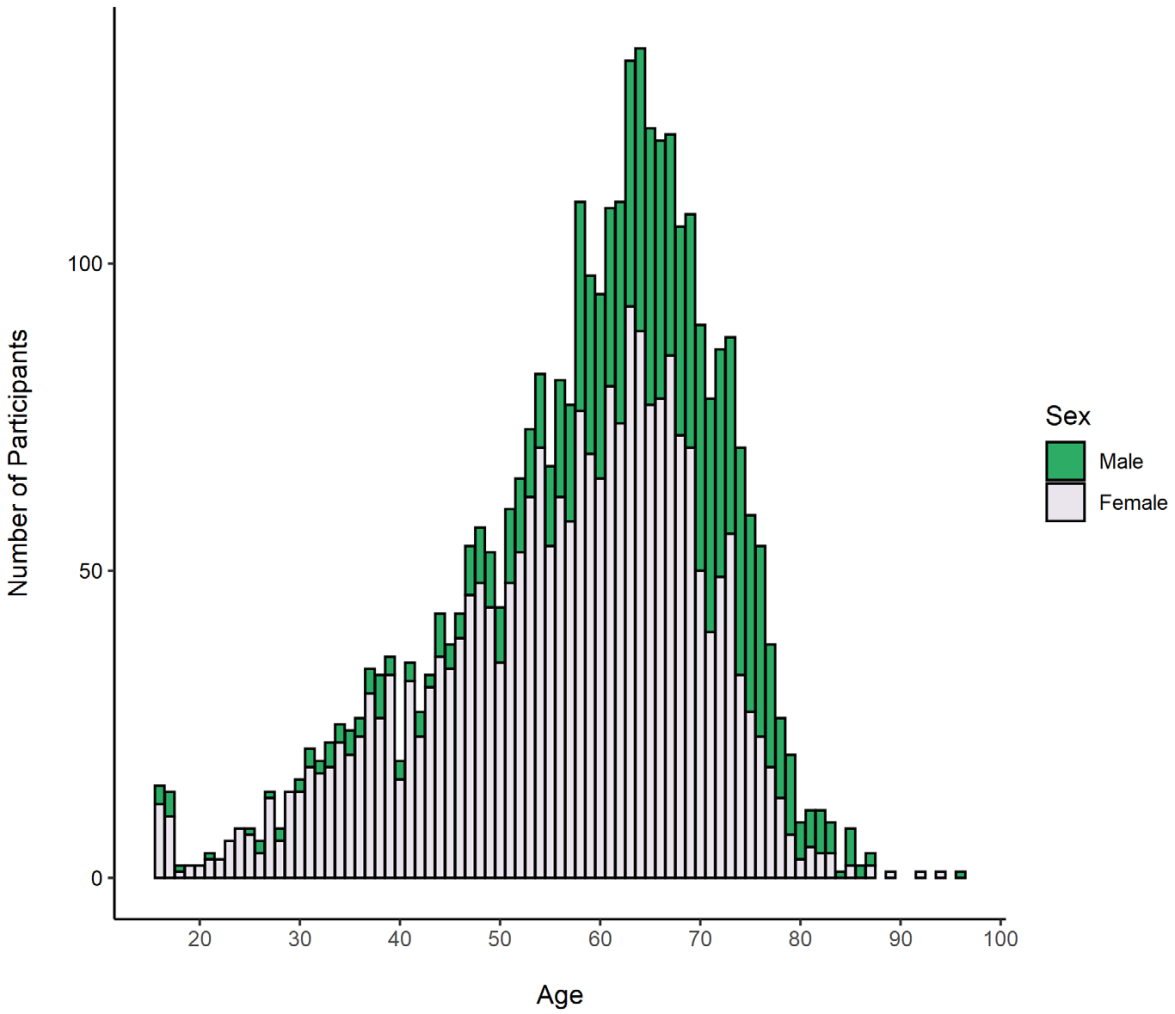
Age distribution of RuralCovidLife participants grouped by sex.

Demographic information for these participants is shown in Table 2. The sample comprised of 2,372 female participants (70.5%) and 979 male participants (29.1%), ranging in age from 16 to 96 years (mean = 58.4, standard deviation [SD] = 13.3). The age distribution of participants by sex is shown in Figure 2.

**Table 2.** Demographic characteristics of the RuralCovidLife sample.

	Mean (SD)
**Age (years)**	58.4 (13.3)
	**N (%)**
**Sex**	
Female	2,372 (70.5)
Male	979 (29.1)
**Gender**	
Female	2,364 (70.2)
Male	975 (29.0)
**Urban/rural classification**	
Remote rural	1,054 (31.3)
Accessible rural	2,099 (62.4)
Remote small towns	52 (1.5)
Non-rural	149 (4.4)
Data unavailable	11 (0.3)
**Ethnicity**	
White	3,137 (93.2)
Non-White	25 (0.7)
Prefer not to answer	27 (0.8)
No response	176 (5.2)
**Education**	
Degree	1,667 (49.5)
No degree	1,485 (44.1)
Prefer not to answer	27 (0.8)
No response	186 (5.5)
**SIMD deciles**	
1 – 2 (most deprived)	20 (0.6)
3 – 4	255 (7.6)
5 – 6	1,060 (31.5)
7 - 8	1,576 (46.8)
9 – 10 (least deprived)	442 (13.1)

SD: standard deviation; SIMD: Scottish Index of Multiple Deprivation Data on Rural-Urban classification for participants who did not provide a valid postcode

The majority of the participants were white (n = 3,137, 93.2%) and lived in less deprived areas (Scottish Index of Multiple Deprivation [SIMD] decile > 5: n = 2,696, 80.1%). Almost half were educated to degree level (n = 1,667, 49.5%).

A total of 7.0% (n = 235) had been told they were at severe risk from COVID-19 and needed to shield themselves. A tenth of participants (10.4%; n = 349) reported that they had or had had COVID-19, whether suspected or confirmed with a test.

Table 3 shows the number of participants by local authority area. The highest proportion of participants was from Aberdeenshire (n = 542, 16.1%); The Aberdeenshire area, the Perth and Kinross area, and the Highland area accounted for 42.3% of participants.

**Table 3.** Responses by local authority areas.

Local authority	N (%)
Aberdeen city	22 (0.7)
Aberdeenshire	542 (16.1)
Angus	292 (8.7)
Argyll and Bute	158 (4.7)
City of Edinburgh	17 (0.5)
Clackmannanshire	20 (0.6)
Dumfries and Galloway	224 (6.7)
Dundee City	< 10
East Ayrshire	22 (0.6)
East Dunbartonshire	23 (0.7)
East Lothian	95 (2.8)
East Renfrewshire	< 10
Falkirk	17 (0.5)
Fife	332 (9.9)
Glasgow City	< 10
Highland	368 (10.9)
Midlothian	37 (1.1)
Moray	83 (2.5)
Na h-Eileanan Siar	55 (1.6)
North Ayrshire	28 (0.8)
North Lanarkshire	18 (0.5)
Orkney Islands	55 (1.6)
Perth and Kinross	515 (15.3)
Renfrewshire	17 (0.5)
Scottish Borders	133 (4.0)
Shetland Islands	53 (1.6)
South Ayrshire	33 (1.0)
South Lanarkshire	88 (2.6)
Stirling	60 (1.8)
West Dunbartonshire	< 10
West Lothian	35 (1.0)

Postcodes were mapped onto the Scottish Government Rural-Urban classification ^9^; most participants lived in accessible rural locations (n = 2,099, 62.4%), with 31.3% (n = 1,054) living in remote rural, and 1.5% (n = 52) living in remote small towns. A subgroup of the sample lived on one of the Scottish islands (n = 290, 8.6%).

Of the total sample, 91% (n = 3058) had full GAD-7 data. Of these, 17.4% (n = 531) scored 10 or above ^22^, evidencing potential clinical anxiety. The median score was 3 (IQR = 7).

Of the total sample, 88% (n = 2973) had full PHQ-9 data. Of these, 19.2% (n = 571) scored 10 or above ^26^, evidencing potential clinical depression. The median score was 3 (IQR = 7).

**Figure 3.**
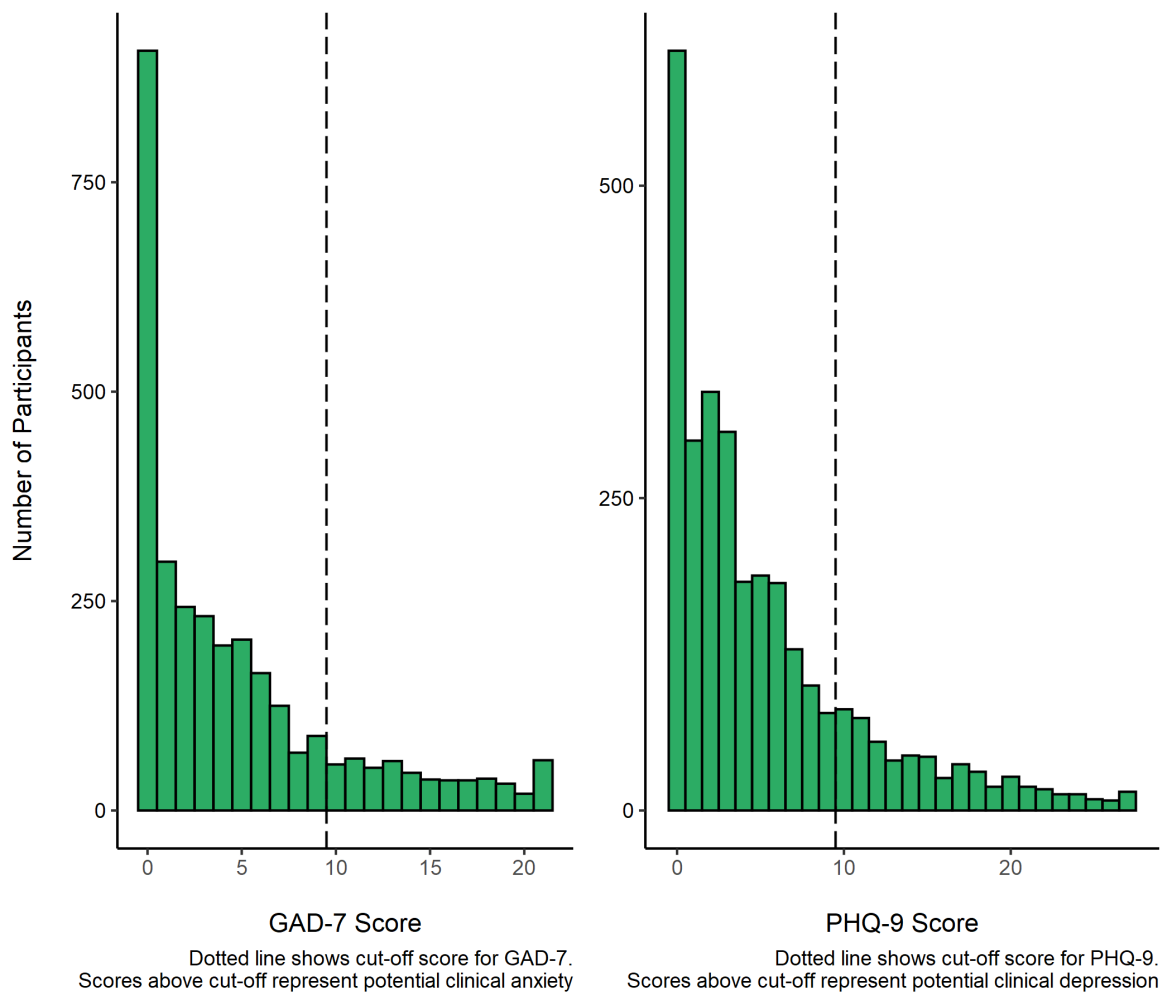
GAD-7 and PHQ-9 scores in RuralCovidLife participants.

Figure 3 shows the distribution of GAD-7 and PHQ-9 scores across participants.

**Figure 4.**
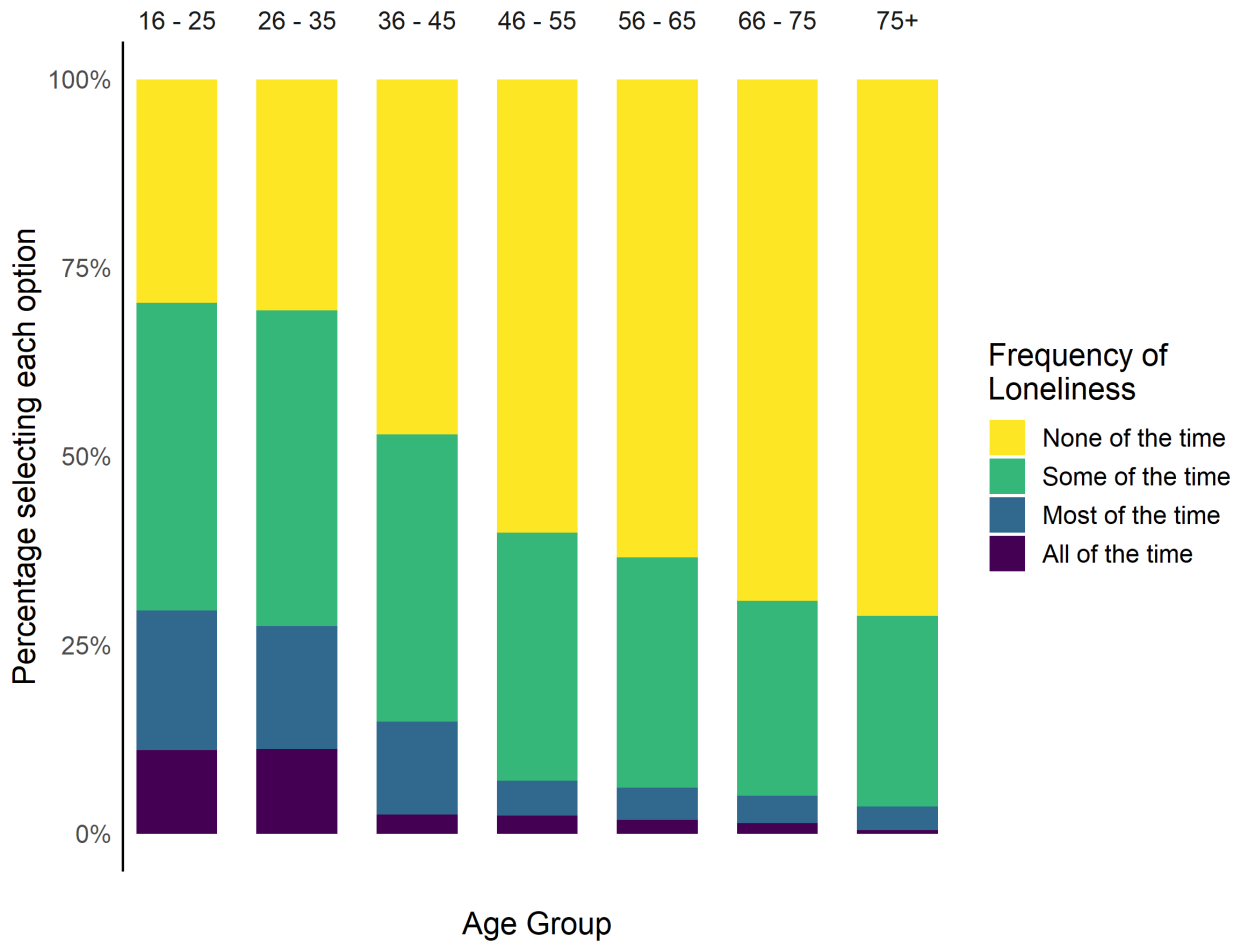
Percentage of participants reporting different frequencies of loneliness.

Overall, 8.1% (n = 264) of participants reported feeling lonely most or all of the time in the past week. Figure 4 shows the percentage of participants reporting frequency of loneliness by age group, after excluding participants who did not answer the question or responded ‘Prefer not to say’ or ‘Don’t know’ (n = 106, 3.2%).

Participants rated overall life satisfaction from 0 (‘Not at all satisfied’) to 10 (‘Extremely satisfied’), with 5 representing ‘Neither satisfied or dissatisfied’. Mean life satisfaction score was 6.14 (SD = 2.32), and 22.9% (n = 742) participants reported low life satisfaction.

Early results were reported and distributed online ^17^, and can be accessed for free on the Generation Scotland website.

## Strengths and limitations

The RuralCovidLife study offers a unique insight into how rural Scottish communities specifically were impacted by the COVID-19 pandemic from the first lockdown in March to autumn 2020. Through individual and small group consultations with people with lived experience in rural communities, the survey incorporated the issues most pertinent to rural Scotland in its design. The resulting dataset is one of the largest COVID-19 studies of the rural population in Scotland. Studying how these communities were differentially affected by the pandemic may be important to implement support in the future. Furthermore, the dataset captures information from a wide range of ages and locations across Scotland.

As RuralCovidLife took advantage of existing research projects within Generation Scotland, different datasets can be linked together. In particular, 523 RuralCovidLife participants were Generation Scotland volunteers and had extensive pre-pandemic data available. This makes it possible to link this subset of RuralCovidLife data not only to pre-pandemic survey data but also, for example, to genetic and health record data. Moreover, through continued health-record linkage in Generation Scotland, it is also possible to examine the prospective impact of the pandemic on health and wellbeing for this subsample. Responses to RuralCovidLife can also be linked to CovidLife data for any participants who took part in both projects, allowing comparison to initial pandemic data in April 2020.

Due to COVID-19 restrictions, the study was conducted remotely and was thus restricted to those with Internet access, which may exclude at-risk groups with poor digital connectivity. However, this may also have allowed participants from more remote areas who would otherwise struggle to participate in research projects to get involved. Additionally, the study is not representative, with the majority of respondents being older and female. Participants were recruited through a mental health network, and a high proportion of participants scored above the cut-off on the GAD-7 and PHQ-9. As such, participants with mental distress may be over-represented. Participants also tended to be from less deprived areas, so the impact of SES on mental health and COVID-19 impact may be underestimated. Very few ethnic minority participants took part, making comparisons based on ethnic background unlikely to be tenable. Finally, recruitment was largely reliant on people who have already expressed an interest in health research, through participation in projects such as SHARE or Generation Scotland. These factors may limit the generalisability of the data.

The data was collected at a relatively early stage of the pandemic, before any COVID-19 vaccines became available. As such, this dataset does not capture uptake or attitudes towards COVID-19 vaccines. However, vaccine uptake in the sub-sample who are also part of Generation Scotland (15.6%) can be assessed by examining linked data.

## Conclusions

RuralCovidLife provides a valuable resource for investigating the effects of the COVID-19 pandemic on people in rural Scotland, built from a collaboration between researchers and members of those communities. While the pandemic has been challenging for many people, the remoteness of rural areas and their reliance on tourism means that rural communities face particular challenges. RuralCovidLife provides data to learn more about how rural communities have responded to the pandemic, and to help these communities recover in the future.

## Data availability

### Underlying data

The de-identified RuralCovidLife dataset is available to researchers in the UK and internationally. Researchers can apply for access to the RuralCovidLife data on the Generation Scotland website. The application form and further details of how to apply can be found on the Generation Scotland website.

### Extended data

Zenodo: Extended data for “RuralCovidLife: Study protocol and description of the data”, https://doi.org/10.5281/zenodo.5589038 ^20^

This project contains the following extended data:

- 2021-10-16 RuralCovidLife Questionnaire GENERAL PUBLIC.docx
- 2021-10-16 RuralCovidlife Questionnaire PERSONALISED LINK.docx
- 2021-10-16 RuralCovidLife VIS & Consent.docx
- CovidLife_Access_Request_Form_V3.1_March _2021.docx
- Generation_Scotland_Access_Request_Form_V1.2_March_2021.docx

Data are available under the terms of the Creative Commons Attribution 4.0 International license (CC-BY 4.0).
