# Supplementary material for: RuralCovidLife: A new resource for the impact of the pandemic on rural Scotland
Source: Wellcome Open Res. 2022 May 23;6:317. Originally published 2021 Nov 23. [Version 2] doi: 10.12688/wellcomeopenres.17325.2 (PMC11079587; doi:10.12688/wellcomeopenres.17325.2)
Supplement: Peer review history

# Reviewer report: Reviewer response for version 2

**DOI:** 10.21956/wellcomeopenres.19832.r78556

**Published:** 2024-05-08

**Authors:** Clifford Stevenson

**Affiliations:** 1 Nottingham Trent University, Nottingham, England, UK

RuralCovidLife is a unique survey of the experiences of 3,365 rural residents of Scotland during the pandemic. This was part of a broader set of surveys designed to capture the effects of the pandemic and the accompanying social restrictions in their early stages in Scotland. It used public consultation and PPIE to adapt the CovidLife survey to capture many of the specific challenges and emergent reactions of the rural population to the pandemic. While it is formed of several subsamples from different sources, it includes a subsample of the Generation Scotland panel, which potentially allows linkage to healthcare data. At the time it provided a valuable too to identify and evidence the emerging needs of the rural communities in Scotland during the pandemic and now it affords secondary analyses of the relationships between the variables captured. 

 In addition to the timeliness of the research, one of the strengths of the work lies in the coproduced nature of the questionnaire. This was no mean feat during the pandemic and the authors did well to manage this so effectively. There is some good detail provided on the involvement of the informants and the PPIE panel in the identification of relevant issues. Some further detail on who these 14 contacts and 7 PPIE members were, in terms of their demographics, location etc., would be useful (if it was possible to do this without breaching anonymity). 

 An admitted weakness to the dataset is that it is not representative of the broader rural population. I wondered if it would be useful for the authors to include the census data for the demographics of the rural population of Scotland (age, gender, ethnicity etc) to show where these deviations occur. Likewise it might be useful to compare the geographical distribution of participants to that of the actual rural population of Scotland. Where the sample departs substantially from census figures (e.g. gender) it would be useful to have the authors' thoughts on why this might be the case. 

 The dataset is also limited by lack of representation of minorities. The bulk of research indicates that socially and economically vulnerable groups fared worse during the pandemic and it would be useful to able to compare how these groups experienced the pandemic in rural areas. If possible, a bit more detail on the socioeconomic characteristics of the sample (e.g. employment status) would be useful along with the current reports of loneliness and mental health.

Are sufficient details of methods and materials provided to allow replication by others?

Yes

Is the rationale for creating the dataset(s) clearly described?

Yes

Are the datasets clearly presented in a useable and accessible format?

Yes

Are the protocols appropriate and is the work technically sound?

Yes

Reviewer Expertise:

Social Psychology; social relationships and health; health and wellbeing among marginalised communities; local community belonging and resilience.

I confirm that I have read this submission and believe that I have an appropriate level of expertise to confirm that it is of an acceptable scientific standard.

# Reviewer report: Reviewer response for version 2

**DOI:** 10.21956/wellcomeopenres.19832.r50692

**Published:** 2022-07-12

**Authors:** Holly Hope

**Affiliations:** 1 Centre for Women’s Mental Health, School of Health Sciences, University of Manchester, Manchester, UK

Thank you for clarifying the objectives, and making minor changes.

 As a data note, it will make an excellent reference for future researchers who use this data to examine pandemic effects on the rural population. It clearly describes the data in the study and how it was collected.

 The interoperability of this dataset with other available datasets and how to access and license the data are clearly described. This will ensure the academic community can re-use these data to understand how pandemic affected populations in rural locations, a group that are too often underrepresented in contemporary research.

Are sufficient details of methods and materials provided to allow replication by others?

Partly

Is the rationale for creating the dataset(s) clearly described?

Yes

Are the datasets clearly presented in a useable and accessible format?

Partly

Are the protocols appropriate and is the work technically sound?

Partly

Reviewer Expertise:

Psychiatric epidemiology, impact of COVID, behavioural science.

I confirm that I have read this submission and believe that I have an appropriate level of expertise to confirm that it is of an acceptable scientific standard.

# Reviewer report: Reviewer response for version 1

**DOI:** 10.21956/wellcomeopenres.19155.r49908

**Published:** 2022-05-11

**Authors:** Holly Hope

**Affiliations:** 1 Centre for Women’s Mental Health, School of Health Sciences, University of Manchester, Manchester, UK

This is a really interesting piece of research that describes the impact of COVID on rural Scotland.

 Here are my main thoughts, that need to be addressed prior to indexing:

**Introduction:**

- Lacks a clear set of objectives as to what this paper does.
- I do not see how this is a study protocol as it has no objectives, and the data is already collected.

**Results:**

- Rural Scotland ~900,000 people, this sample is only ~3000, what measures were taken to address potential sources of selection bias, for example are any of the studies you recruited from use population based sampling?
- Minor - some typos throughout, need another review and those removed, see ethic section for an example. 
- Results; "Following data cleaning, 1,270 participants were included, leaving 3,365 participants in the final dataset."
  Should this read :
  "Following data cleaning, 1,270 participants were **excluded**, leaving 3,365 participants in the final dataset."

**Discussion:**

- "the study is not representative, with the majority of respondents being older and female. Participants were recruited through a mental health network, and a high proportion of participants scored above cut-off on the GAD-7 and PHQ-9. As such, participants with mental distress may be over-represented."
  I would also suggest that the absence of rural communities from more deprived areas may also underestimate mental health risk.
- At any time, 1 in 5 women and 1 in 8 men report a mental illness. apms-2014-full-rpt.pdf (publishing.service.gov.uk). There is considerable evidence that women's mental health was most affected by COVID restrictions. Please see Mental health before and during the COVID-19 pandemic: a longitudinal probability sample survey of the UK population - PubMed (nih.gov) and Mental health responses to the COVID-19 pandemic: a latent class trajectory analysis using longitudinal UK data - PubMed (nih.gov).
  Therefore, the overall rate of ~1 in 5 suggests that poor mental health has increased for either men or women, the discussion lacks any discussion for why that might be.
- Have the authors considered weighting the GAD or PHQ estimates to give a more accurate overall estimate, or considered reporting scores for men and women (in age bands) separately, to give a more nuanced overview of mental health (and therefore a little more to discuss in the discussion)? 
- Why do we see higher rates of loneliness among young people in rural Scotland than older people -  are these findings replicated in GAD and PHQ (I suspect they are) and does this have any policy implications?
- The possibility of linkages to other datasets is useful, but it would be helpful for the reader if the authors could envisage the types of projects that are possible with this dataset and any planned future analyses in the discussion.

Are sufficient details of methods and materials provided to allow replication by others?

Partly

Is the rationale for creating the dataset(s) clearly described?

Yes

Are the datasets clearly presented in a useable and accessible format?

Partly

Are the protocols appropriate and is the work technically sound?

Partly

Reviewer Expertise:

Psychiatric epidemiology, impact of COVID, behavioural science.

I confirm that I have read this submission and believe that I have an appropriate level of expertise to state that I do not consider it to be of an acceptable scientific standard, for reasons outlined above.

# Response

**Published:** 2022-05-16

**Authors:** Charlotte Huggins

**Affiliations:** University of Edinburgh, UK

Thank you for your thoughtful and detailed review. Please find below responses to your key points. These have been addressed as best as possible in the newest version of the manuscript.

*Introduction:*

*• Lacks a clear set of objectives as to what this paper does.*

*• I do not see how this is a study protocol as it has no objectives, and the data is already collected.*

 We agree that the objective of the paper was not clearly enough stated in the previous draft, and this has caused some confusion. We would like to stress that this is a Wellcome Open Research ‘Data Note’. This means it makes no interpretation of the collected data, and is instead intended only to describe the data resource to make it available for other research use.

 To clarify this, we have changed the title of the paper to:

 “RuralCovidLife: A new resource for the impact of the pandemic in rural Scotland”

 We have also added a small line to the introduction, to clarify the purpose of the paper, see line 208 - 210:

 “This is a data note intended to act as an information resource for researchers, and does not make any interpretations of the data or its implications.”

*Results:*

*• Rural Scotland ~900,000 people, this sample is only ~3000, what measures were taken to address potential sources of selection bias, for example are any of the studies you recruited from use population based sampling?*

 Unfortunately, the data was collected through convenience sampling, due to the need for a quick turnaround to collect relevant data during the early phase of the pandemic while conducting the participatory element of the study. This limitation has been clarified in the methods section, see line 310 – 311:

 “Recruitment was conducted through convenience sampling, so sample may not be representative.”

*• Minor - some typos throughout, need another review and those removed, see ethic section for an example.*

 Thank you, the paper has been carefully proofread to identify any outstanding issues, and these have been removed.

*• Results; "Following data cleaning, 1,270 participants were included, leaving 3,365 participants in the final dataset."*

*Should this read :*

*"Following data cleaning, 1,270 participants were excluded, leaving 3,365 participants in the final dataset."*

 Thank you for pointing out this error. This has been amended.

*Discussion:*

*• "the study is not representative, with the majority of respondents being older and female. Participants were recruited through a mental health network, and a high proportion of participants scored above cut-off on the GAD-7 and PHQ-9. As such, participants with mental distress may be over-represented."*

*I would also suggest that the absence of rural communities from more deprived areas may also underestimate mental health risk.*

 Thank you for highlighting this, we have added a statement acknowledging these important issue, see line 466 – 467:

 “Participants also tended to be from less deprived areas, so the impact of SES on mental health and COVID-19 impact may be underestimated”

*• At any time, 1 in 5 women and 1 in 8 men report a mental illness. apms-2014-full-rpt.pdf (publishing.service.gov.uk). There is considerable evidence that women's mental health was most affected by COVID restrictions. Please see Mental health before and during the COVID-19 pandemic: a longitudinal probability sample survey of the UK population - PubMed (nih.gov) and Mental health responses to the COVID-19 pandemic: a latent class trajectory analysis using longitudinal UK data - PubMed (nih.gov).*

*Therefore, the overall rate of ~1 in 5 suggests that poor mental health has increased for either men or women, the discussion lacks any discussion for why that might be.*

 We agree this is an important issue. However, Wellcome Open Research Data Notes are not intended to give interpretations of data. It is instead intended as a relatively neutral description of the data and how it was collected, to inform future researchers using the resource. As such, we do not believe it is appropriate to make such assertions in this paper. This would be an interesting question for a researcher using the resource to investigate.

*• Have the authors considered weighting the GAD or PHQ estimates to give a more accurate overall estimate, or considered reporting scores for men and women (in age bands) separately, to give a more nuanced overview of mental health (and therefore a little more to discuss in the discussion)?*

*• Why do we see higher rates of loneliness among young people in rural Scotland than older people -  are these findings replicated in GAD and PHQ (I suspect they are) and does this have any policy implications?*

 We agree the above two points are important considerations that merit further careful discussion and analysis. However, the current submission is intended as a descriptive resource, rather than a scientific analysis of the data. We therefore believe such discussion and interpretation of the results would not be appropriate for a data note.
